# Calycosin stimulates the proliferation of endothelial cells, but not breast cancer cells, via a feedback loop involving RP11-65M17.3, BRIP1 and ERα

**DOI:** 10.18632/aging.202641

**Published:** 2021-03-01

**Authors:** Yong Wang, Wei Xie, Mengyue Hou, Jing Tian, Xing Zhang, Qianyao Ren, Yue Huang, Jian Chen

**Affiliations:** 1 Key Laboratory of Tumor Immunology and Microenvironmental Regulation of Guangxi, Guilin Medical University, Guilin 541004, Guangxi, China; 2 Department of Physiology, Guilin Medical University, Guilin 541004, Guangxi, China; 3 Department of Breast and Thyroid Surgery, First Affiliated Hospital of Guilin Medical University, Guilin 541001, Guangxi, China

**Keywords:** calycosin, postmenopausal, endothelial cells, breast cancer, RP11-65M17.3

## Abstract

It is widely accepted that estrogen can be replaced by phytoestrogens to treat postmenopausal cardiovascular disease and possibly decrease the risk of breast cancer. However, few studies have investigated the effects of phytoestrogens on vascular endothelial cells (ECs). In the present study, we show that the phytoestrogen calycosin (20 μM) stimulated the proliferation of ECs (HUVECs and HMEC-1) but inhibited the growth of breast cancer cells (BCCs) expressing ERα (MCF-7 and T47D). Here we provide evidence for the presence of a positive feedback loop between ERα and long noncoding RNA RP11-65M17.3 in both normal and cancer cells, and calycosin stimulated this feedback loop in ECs but decreased RP11-65M17.3 expression in BCCs. Subsequently, the calycosin-induced activation of this loop decreased the expression of the target of BRIP1 (BRCA1 interacting protein C-terminal helicase 1), increased the phosphorylation of Akt and ERK1/2, and finally inhibited the cleavage of PARP-1 in ECs. In nude mice bearing MCF-7 xenografts, calycosin did not stimulate tumor growth as strongly as 17β-estradiol. Together, these results suggest that calycosin promotes the proliferation of ECs, and notable inhibits the growth of BCCs. A possible reason for these results is the involvement of a feedback loop between ERα and RP11-65M17.3.

## INTRODUCTION

According to the World Bank, the total population of postmenopausal women worldwide is predicted to reach 1200 million by 2030, and the proportion of these women living in the developing world will increase to 76% [1, 2]. Decreased levels of sex hormones in the blood are thought to be responsible for postmenopausal syndrome, which is mainly caused by cessation of ovarian function and decline in steroid and peptide hormone secretion. Studies have shown that sex hormones such as estrogen participate in regulating the function of endothelial cells (ECs), and endothelial dysfunction is associated with postmenopausal cardiovascular diseases, including coronary artery disease, atherosclerosis, and stroke [3]. Estrogen accelerates endothelial regrowth, which favors vascular healing, and it prevents EC apoptosis [4, 5]. Therefore, the induction of endothelial dysfunction by postmenopausal syndrome could be significantly ameliorated by exogenous estrogen. However, long-term treatment with estrogen is believed to be associated with the occurrence of cancers, such as endometrial cancer, ovarian cancer, and especially ER(+) breast cancer, and the US Food and Drug Administration considers steroidal estrogens to be human carcinogens [6]. It is imperative to develop safer and more efficient alternatives to hormone replacement therapy (HRT) for the treatment of postmenopausal syndrome.

Interestingly, Li et al. reported that, when treating postmenopausal syndrome, isoflavone phytoestrogens markedly improve flow-mediated dilation only in postmenopausal women with impaired endothelial function, suggesting that isoflavones may exert coronary benefits by improving endothelial function [7]. More importantly, isoflavones were indicated to promote the proliferation of breast cancer cells (BCCs) to a lesser extent than 17β-estradiol (E_2_), and isoflavones may be associated with a lower risk of postmenopausal breast cancer [8–10]. Together, these data suggest that isoflavones may be beneficial for treating postmenopausal cardiovascular disease by regulating the function of ECs.

Calycosin, a major isoflavonoid in *Radix Astragali Mongolici*, was demonstrated to successfully induce angiogenesis in zebrafish and human umbilical vein ECs (HUVECs) via estrogen receptor (ER)-mediated activation of the MAPK signaling pathway [11]. Consistent with this concept, in our previous study, calycosin was found to upregulate the level of ERK1/2 and stimulate a dramatic increase in uterine weight in ovariectomized (OVX) rats [12]. However, some reported that calycosin alone elicited no response in cultured ECs. Such conflicting results make it difficult to establish whether calycosin exerts protective effects against cardiovascular disease by promoting the growth of ECs, and the involved mechanisms remain to be determined. Therefore, in this study, we focused on the calycosin-induced regulation of EC proliferation *in vitro* and *in vivo*. Additionally, two estrogen receptor (ER)-positive breast cancer cell lines (MCF-7 and T47D) were included in this study to examine whether the effect on proliferation was specific to normal epithelial cell lines.

Recently, lncRNAs (long noncoding RNAs) have emerged as new regulators of cellular processes, such as proliferation, differentiation and survival [13]. It was reported that estrogen could induce the expression of lncRNAs, while lncRNAs were proven to be coactivators of ERα, whose signaling is central to estrogen-dependent cells [14, 15]. Conversely, ERα can stimulate or repress lncRNA transcription. In a preliminary screening of lncRNAs in HUVECs, we showed that calycosin upregulated the expression levels of several lncRNAs, and RP11-65M17.3 was found to be upregulated in ERα-positive HUVECs. Thus, to explore how calycosin affects ERα-positive ECs and BCCs, we examined whether an interaction exists between RP11-65M17.3 and the ERα loop, through which the compound affects the proliferation of ECs and BCCs. Additionally, in HUVECs and HMEC-1 cells, the target of RP11-65M17.3 was confirmed to be BRIP1 (BRCA1 interacting protein C-terminal helicase 1) by a luciferase reporter assay. BRIP1 is a regulatory subunit B (alpha isoform) of protein phosphatase 2A (PP2A) and negatively controls cell growth and division in mammalian cells [16–18]. Therefore, we also assumed the involvement of the RP11-65M17.3-mediated BRIP1 pathway in the effect of calycosin on EC proliferation.

## RESULTS

### Dual role of calycosin in regulating the proliferation of ECs and BCCs

**Figure 1:**
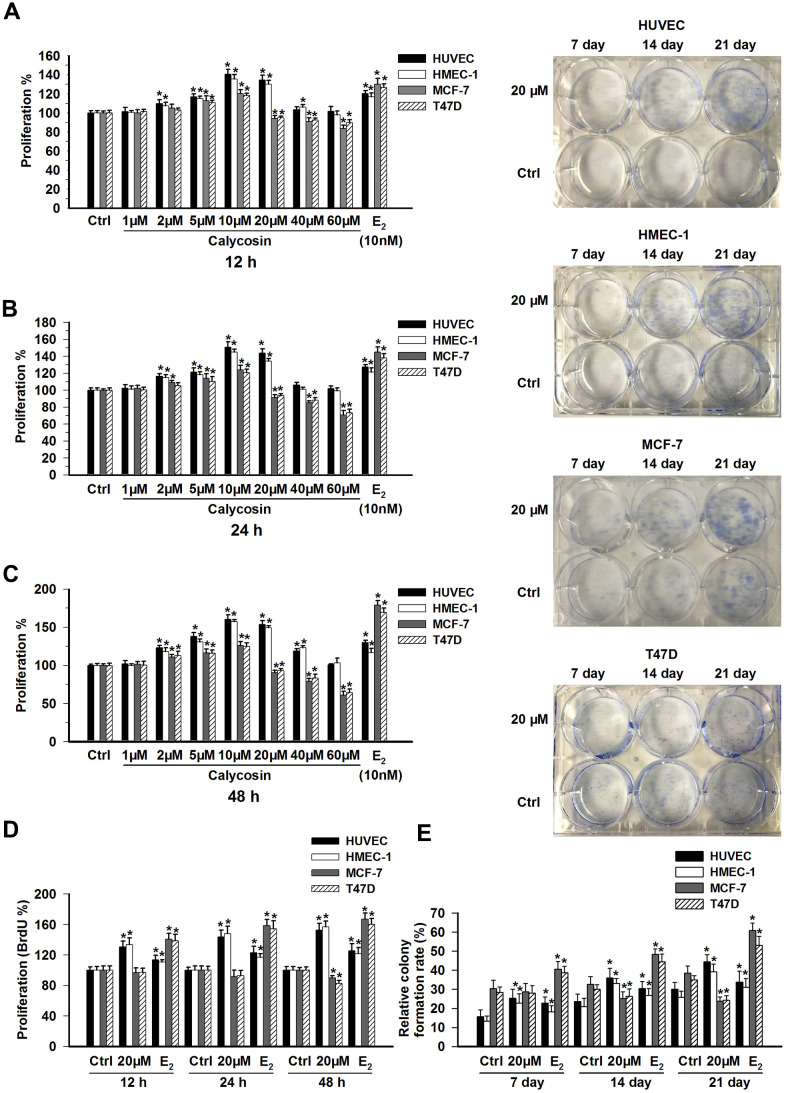
**Effects of calycosin on the proliferation of ECs and BCCs.** (**A**–**C**) HUVECs, HMEC-1 cells, MCF-7 cells and T47D cells were treated with calycosin (1-60 μM) or E_2_ (10 nM) for 12, 24, or 48 h. Cell proliferation was determined using a CCK-8 assay. (**D**) HUVECs, HMEC-1 cells, MCF-7 cells and T47D cells were treated with calycosin (20 μM) or E_2_ (10 nM) for 12, 24, or 48 h. Cell proliferation was determined using the BrdU assay. (**E**) For the colony formation assays, after treatment with calycosin (20 μM) or E_2_ (10 nM) for 7, 14, and 21 days, the numbers of cell colonies were counted. The results are from three independent experiments performed in triplicate. *p < 0.05 vs. control (0 μM).

We examined the proliferation of HUVECs, HMEC-1, MCF-7 and T47D cells treated with different concentrations of calycosin. When exposed to calycosin at lower concentrations (1 μM to 10 μM), a dose-dependent stimulation of proliferation was observed in both the endothelial and breast cancer cell lines, and this effect was more prominent in the HUVECs and HMEC-1 cells, as shown in Figure 1A–1C (*p* < 0.05). When the concentration of calycosin was further increased to 60 μM, growth inhibition was observed in the MCF-7 and T47D cells (*p* < 0.05). Interestingly, although the strength of the effect of calycosin on the proliferation of the endothelial cell lines was gradually reduced, no inhibitory effect was observed, even at the highest concentration of 60 μM. Notably, when the calycosin concentration was 20 μM, calycosin still promoted the growth of the HUVECs and HMEC-1 cells but started to inhibit the proliferation of the MCF-7 and T47D cells (*p* < 0.05). Consistent with the CCK8 assay results, increased BrdU and CFE (clone formation efficiency) incorporation, which was comparable to that of the control, was only observed in the HUVEC and HMEC-1 endothelial cell lines following treatment with 20 μM calycosin (Figure 1D, 1E; *p* < 0.05). As a positive control, we treated the four cell lines with 10 nM E_2_ and observed a marked increase in the proliferation of all the cell lines (*p* < 0.05), especially in the proliferation of the two breast cancer cell lines.

### Involvement of the RP11-65M17.3-ERα feedback loop in the proliferation of ECs and BCCs

**Figure 2:**
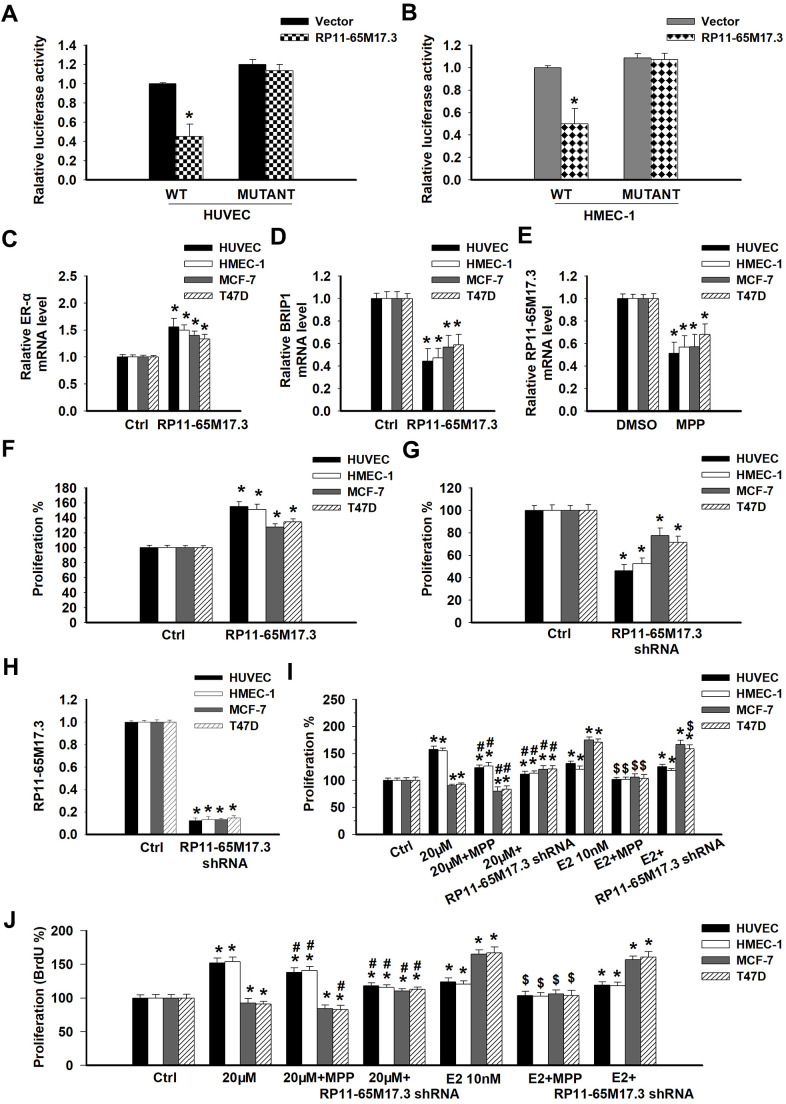
**The association among RP11-65M17.3, BRIP1 and ERα in ECs and BCCs and the involvement of RP11-65M17.3 and ERα in calycosin mediated regulation of cell proliferation.** (**A**, **B**) A luciferase reporter assay was performed to determine whether BRIP1 is the downstream target of RP11-65M17.3 in HUVECs and HMEC-1 cells. *p < 0.05 vs. vector. (**C**–**E**) The existence of a positive feedback loop between RP11-65M17.3 and ERα was demonstrated in HUVECs, HMEC-1 cells, MCF-7 cells and T47D cells. The cells were treated with the plasmid construct pCDNA3.1-RP11-65M17.3 or the ERα antagonist MPP. The mRNA expression levels of ERα, BRIP1, and RP11-65M17.3 were determined by qRT-PCR, and β-actin was used as the internal control. (**F**, **G**) The overexpression of RP11-65M17.3 stimulated cell proliferation, while the knockdown of RP11-65M17.3 inhibited proliferation, as detected by CCK8 assay. *p < 0.05 vs. control. (**H**) Pretreatment with pCDNA3.1-RP11-65M17.3 shRNA downregulated the expression of RP11-65M17.3 in HUVECs, HMEC-1 cells, MCF-7 cells and T47D cells. *p < 0.05 vs. control. (**I**, **J**) The four cell lines were then treated for 48 h with 20 μM calycosin, 20 μM calycosin plus MPP, 20 μM calycosin plus RP11-65M17.3 shRNA, 10 nM E_2_, 10 nM E_2_ plus MPP, or 10 nM E_2_ plus RP11-65M17.3 shRNA. Cell proliferation was analyzed using the CCK-8 assay and BrdU assay. Representative data from three independent experiments are shown. *p < 0.05 vs. control (0 μM); ^#^*p* < 0.05 vs. 20 μM calycosin alone; ^$^*p* < 0.05 vs. 10 nM E_2_ alone.

To explore the potential mechanisms by which calycosin regulates the proliferation of ECs or BCCs, the lncRNA levels were measured via microarray following treatment with the compound. Calycosin significantly upregulated 10 lncRNAs in the HUVECs (fold change >2; *p* < 0.05) and had the greatest effect on RP11-65M17.3 expression (Table 1). Gene function analysis further indicated that the significant upregulation of these lncRNAs was related to the regulation of cellular growth, proliferation and death. We focused on BRIP1 as a functional target of RP11-65M17.3, which was confirmed in HUVECs and HMEC-1 cells by luciferase reporter assays (Figure 2A, 2B; p < 0.05). Overexpressing RP11-65M17.3 increased ERα expression and decreased BRIP1 expression in not only the ECs (HUVECs and HMEC-1 cells) but also the BCCs (MCF-7 and T47D cells), as shown in Figure 2C, 2D (*p* < 0.05). On the other hand, RP11-65M17.3 expression was, in turn, dependent on ERα expression because ERα inhibition with MPP (methylpiperidino pyrazole) decreased the RP11-65M17.3 levels in all four cell lines (Figure 2E; *p* < 0.05). Additionally, the overexpression of RP11-65M17.3 stimulated the proliferation of the HUVECs and HMEC-1, MCF-7 and T47D cells, whereas the pretreatment with RP11-65M17.3 shRNA inhibited the growth of the four cell lines (Figure 2F, 2G; *p* < 0.05). These data present evidence supporting the existence of a positive feedback loop between RP11-65M17.3 and ERα in both normal ECs and BCCs that may play an important role in cell growth regulation.

**Table 1:** The effects of calycosin treatment on lncRNA profiles in HUVECs.

**NO.**	***P*. Value**	**Gene_symbol**	**Log 2 ratio** **HUVEC**
1	4.10812E-06	RP11-65M17.3	6.41
2	3.28138E-05	FTX|FTX|FTX	5.92
3	3.63562E-05	AC023469.2	5.62
4	1.76564E-05	RP4-622L5.7	5.15
5	1.43090E-04	THRB-AS1	4.48
6	1.59763E-04	RP11-277A4.4	4.43
7	3.71157E-05	RP1-60N8.1	3.57
8	9.74799E-05	AC010525.4	3.31
9	2.55835E-05	CTD-3224K15.2	2.82
10	9.90277E-06	AC108868.5	2.41

### Involvement of RP11-65M17.3 and ERα in calycosin-regulated cell proliferation in ECs and BCCs

To determine whether the effects of calycosin on proliferation are associated with RP11-65M17.3 and ERα, we treated HUVECs and HMEC-1, MCF-7 and T47D cells with 20 μM calycosin with or without an inhibitor. As shown in Figure 2H, pretreatment with RP11-65M17.3 shRNA significantly reduced the RP11-65M17.3 levels in all four cell lines (*p* < 0.05). This reduction blocked the proliferative effects of calycosin on ECs (HUVECs and HMEC-1 cells) and the antiproliferative effects on BCCs (MCF-7 and T47D cells) (Figure 2I, 2J; *p* < 0.05 vs. 20 μM calycosin alone). Furthermore, the calycosin-induced proliferative effects on ECs could be partially blocked by adding MPP, whereas the inhibitory effects on BCCs were further enhanced (*p* < 0.05 vs. 20 μM calycosin alone). These results indicate the particular involvement of an RP11-65M17.3-ERα positive feedback loop in the calycosin-induced regulation of ERα-positive EC and ERα-positive BCC proliferation but the modulation of this loop was opposite. The E_2_-induced proliferative activity in all four cell lines was inhibited by only MPP (*p* < 0.05 vs. 10 nM E_2_ alone), and it was not sensitive to treatment with RP11-65M17.3 shRNA, suggesting that, instead of RP11-65M17.3, increased expression of ERα by E_2_ contributes to cell survival in these cell types.

### Activation of the positive feedback loop between RP11-65M17.3 and ERα in ECs by calycosin

Next, we explored the calycosin-mediated regulation of RP11-65M17.3, ERα and BRIP1 at the mRNA and protein levels in ERα-positive cells. As shown in Figure 3A–3G, compared with the control treatment, calycosin significantly increased the expression of ERα and RP11-65M17.3 in the HUVECs and HMEC-1 cells, BRIP1 expression was reduced in a dose- and time-dependent manner, and the protein and mRNA expression levels reached their maximum changes at a calycosin concentration of 20 μM at 48 h (*p* < 0.05). The same treatment led to opposite responses in the MCF-7 and T47D cells, including decreased RP11-65M17.3 expression and increased BRIP1 expression, although ERα expression was still increased (*p* < 0.05). Consistent with these cell type-specific effects, pretreating cells with RP11-65M17.3 shRNA attenuated the ERα upregulation and BRIP1 downregulation induced by calycosin in HUVECs and HMEC-1 cells, but not in the MCF-7 or T47D cells (*p* < 0.05 vs. 20 μM calycosin alone) (Figure 3H–3L). Pretreatment with MPP also attenuated the calycosin-induced upregulation of RP11-65M17.3 and downregulation of BRIP1 in the HUVECs and HMEC-1 cells (*p* < 0.05 vs. 20 μM calycosin alone). Additionally, we found that E_2_ enhanced ERα and RP11-65M17.3 expression in all four cell lines without affecting BRIP1 expression in ECs, and this effect was also attenuated by MPP or RP11-65M17.3 shRNA (*P* < 0.05 vs. 10 nM E_2_ alone) (Figure 3H–3M). These results indicate that calycosin stimulates the positive feedback loop between RP11-65M17.3 and ERα in HUVECs and HMEC-1 cells but not in MCF-7 or T47D BCCs.

**Figure 3:**
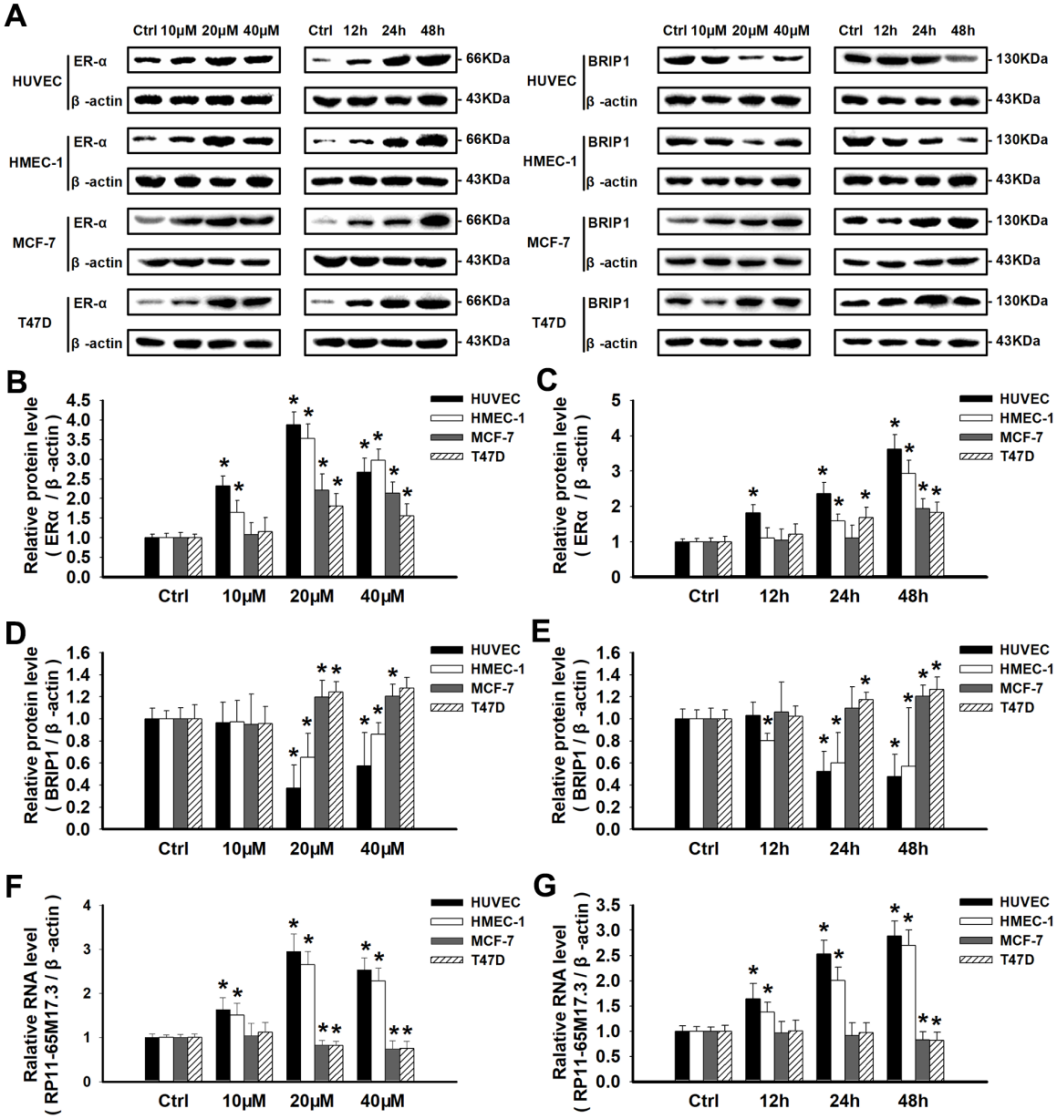
**Calycosin regulated cell proliferation by activating or repressing the RP11-65M17.3-ERα loop.** (**A**–**E**) HUVECs, HMEC-1 cells, MCF-7 cells and T47D cells were treated with calycosin (10, 20 and 40 μM) for 12, 24, or 48 h. Western blotting was used to determine the protein levels of ERα and BRIP1, and β-actin served as the internal control. (**F**, **G**) The levels of RP11-65M17.3 were determined by qRT-PCR and normalized to those of β-actin.

**Figure 3:**
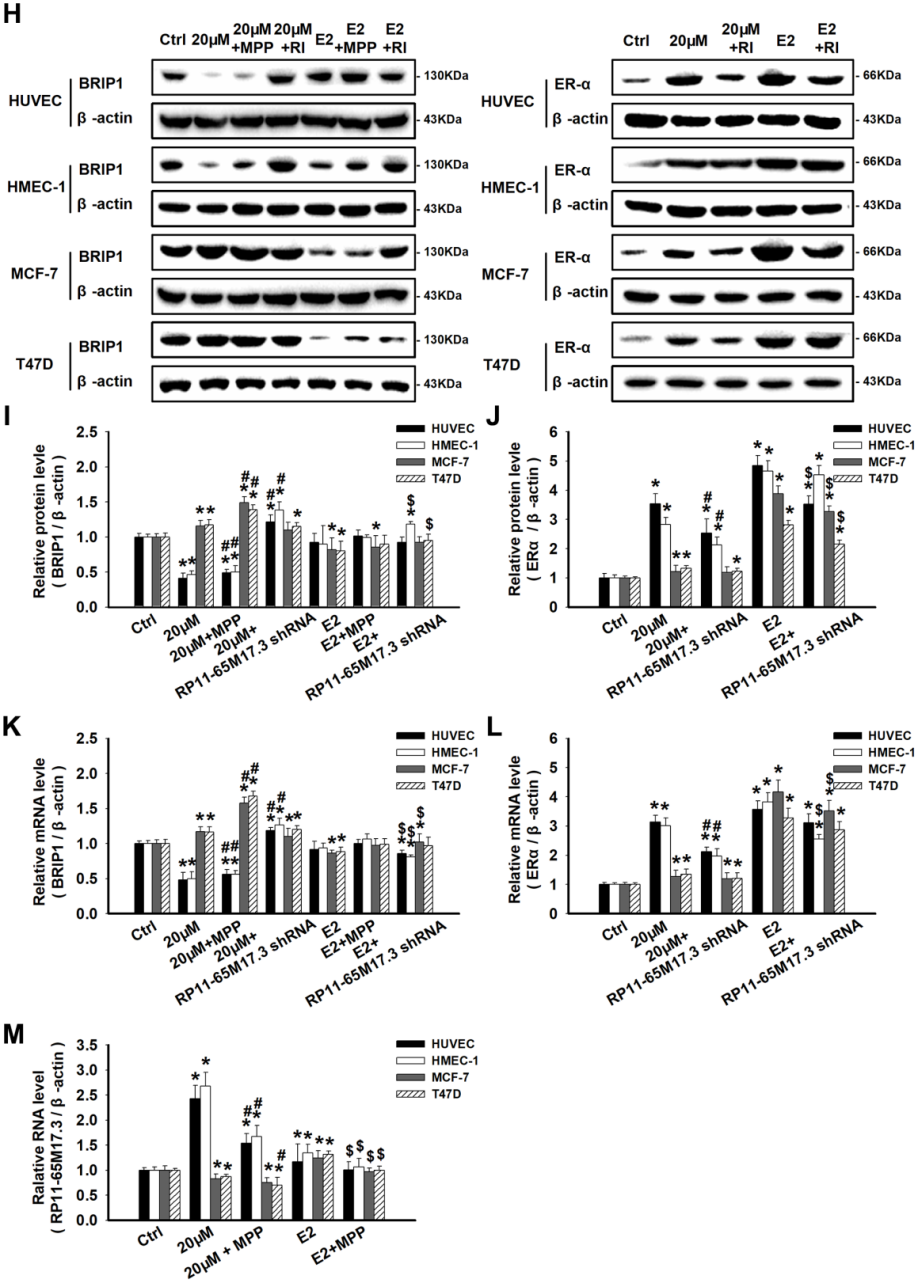
**Calycosin regulated cell proliferation by activating or repressing the RP11-65M17.3-ERα loop.** (**H**–**M**) The effects of calycosin on the RP11-65M17.3-ERα loop were demonstrated by pretreating cells with RP11-65M17.3 shRNA (RI) or MPP. The protein and mRNA expression levels of BRIP1, ERα and RP11-65M17.3 were determined using Western blotting and qRT-PCR. The protein and transcript levels were normalized to the β-actin levels. Representative data from three independent experiments are shown. *p < 0.05 vs. control (0 μM or 0 h); ^#^*p* < 0.05 vs. 20 μM calycosin alone; ^$^*p* < 0.05 vs. 10 nM E_2_ alone.

### Modulation of RP11-65M17.3 and the ERα signaling pathway by calycosin

**Figure 4:**
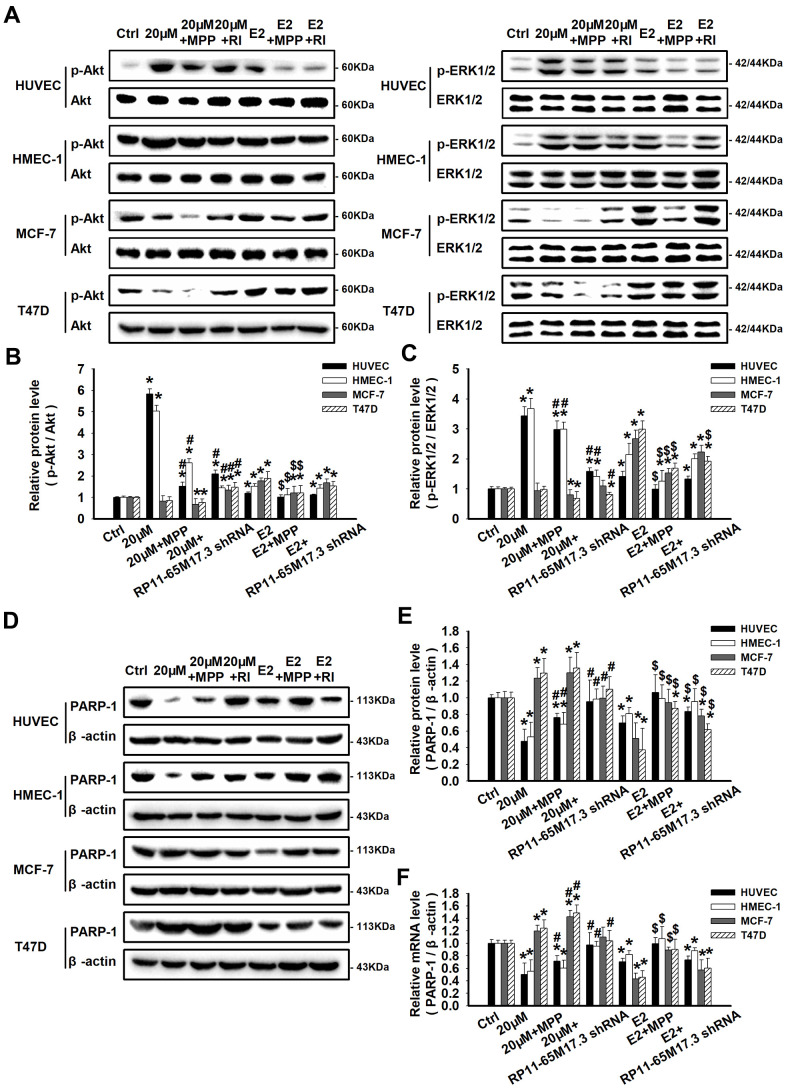
**Regulation of RP11-65M17.3-ERα loop signaling by calycosin in ECs and BCCs.** HUVECs, HMEC-1 cells, MCF-7 cells and T47D cells were pretreated with RP11-65M17.3 shRNA or MPP before incubation with 20 μM calycosin or 10 nM E_2_ alone. (**A**–**C**) The phosphorylation of Akt and ERK1/2 was detected by Western blotting. The corresponding total proteins were used as the internal controls in the same sample. (**D**–**F**) The protein and mRNA expression levels of PARP-1 were determined using Western blotting and qRT-PCR. The expression levels were normalized to those of β-actin. Representative data from three independent experiments are shown. *p < 0.05 vs. control (0 μM); ^#^*p* < 0.05 vs. 20 μM calycosin alone; ^$^*p* < 0.05 vs. 10 nM E_2_ alone.

Previous studies have demonstrated that the expression of BRIP1 is related to the negative regulation of the Akt and ERK1/2 signaling pathways. Generally, ERK1/2 and Akt activation is known to promote cell proliferation, survival and differentiation. Herein, we found that, in calycosin-treated HUVECs and HMEC-1 cells, the levels of phosphorylated ERK1/2 and Akt were significantly enhanced in response to the activation of the RP11-65M17.3-ERα loop and decreased expression of BRIP1 (*p* < 0.05; Figure 4A–4C). Furthermore, it was reported that a decrease in ERK1/2 and Akt phosphorylation contributes to the inhibition of cancer cell growth via the activation of PARP-1. Accordingly, the results showed that elevated phosphorylation of ERK1/2 and Akt was accompanied by inactivation of PARP-1 at both the mRNA and protein levels in ECs, as shown in Figure 4D–4F (*p* < 0.05). Additionally, the protein alterations described above could all be blocked by MPP or RP11-65M17.3 shRNA (*p* < 0.05 vs. 20 μM calycosin alone). In contrast to the ECs, there was an inactivation of ERK1/2 and Akt, and an upregulation of PARP-1 in the calycosin-treated BCCs (*P* < 0.05). It could be proposed that Akt and ERK1/2 act as downstream effectors of the loop between RP11-65M17.3 and ERα, which then stimulate the proliferation of HUVECs and HMEC-1 cells through the inactivation of PARP-1 proteins. E_2_ promoted the phosphorylation of ERK1/2 and Akt and inhibited the expression of PARP-1 in all four cell lines. This effect was mainly blocked by treatment with MPP (*p* < 0.05 vs. 10 nM E_2_ alone).

### Activation of RP11-65M17.3-ERα loop in OVX rats by calycosin

**Figure 5:**
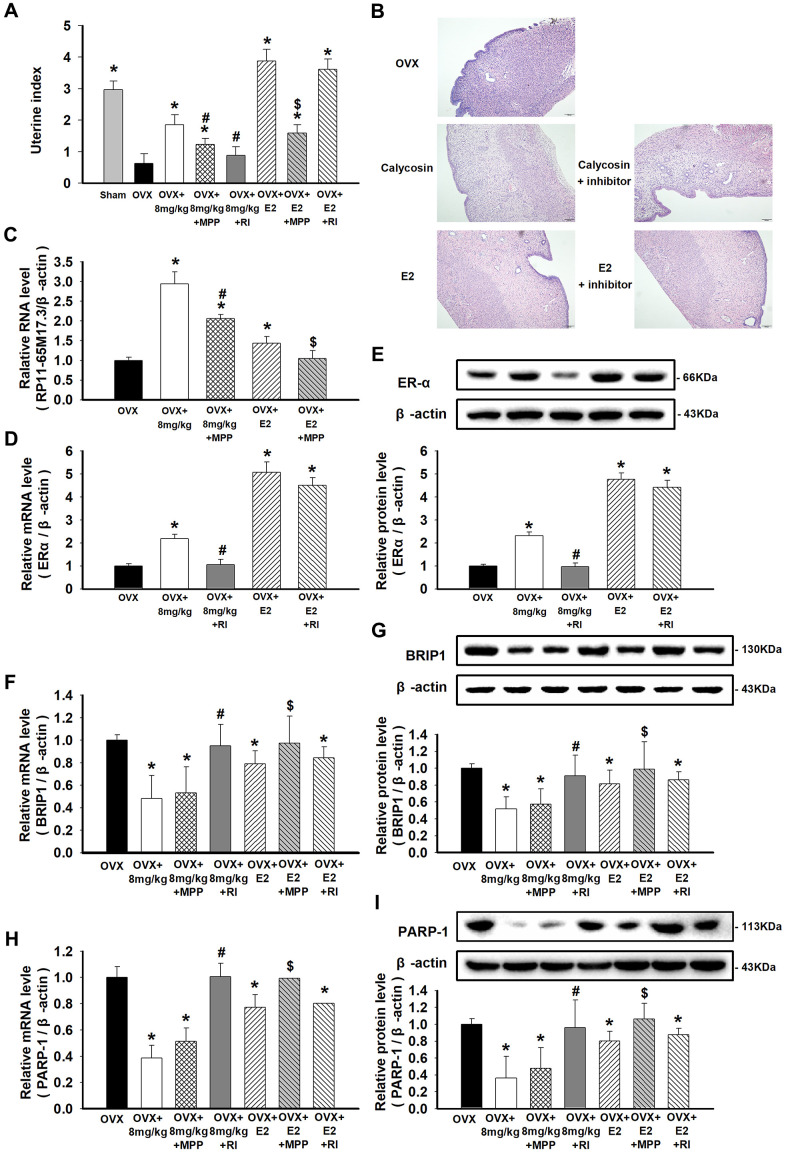
**Effects of calycosin on OVX rats and the activation of the RP11-65M17.3-ERα loop in aortic ECs.** (**A**) OVX rats were treated for 20 days with calycosin (0 or 8 mg/kg), 8 mg/kg calycosin and 5 mg/kg MPP, 8 mg/kg calycosin and RP11-65M17.3 shRNA, 20 μg/kg E_2_, 20 μg/kg E_2_ and 5 mg/kg MPP, 20 μg/kg E_2_ and RP11-65M17.3 shRNA. The uterine index was calculated by the percentage of the uterus weight relative to the body weight. (**B**) The uterine tissues were stained with HE. (**C**–**I**) The levels of RP11-65M17.3, ERα, BRIP1 and PARP-1 in aortic ECs were determined using qRT-PCR or Western blotting. Representative data from three independent experiments are shown. *p < 0.05 vs. OVX; ^#^*p* < 0.05 vs. 8 mg/kg calycosin; ^$^*p* < 0.05 vs. 20 μg/kg E_2_.

The uterine index was significantly decreased in the OVX rats compared with the sham-treated rats (*p* < 0.05), suggesting that the OVX model was successfully established. Next, calycosin exerted a trophic effect on the uterus similar to the effect exerted by estrogen, manifesting as an increase in the uterine index in the OVX rats (*p* < 0.05). Furthermore, morphological analysis revealed the relationship between treatment with calycosin and E_2_ and increased proliferation of endometrial cells in the OVX rats (Figure 5A, 5B). In aortic ECs, calycosin upregulated RP11-65M17.3 and ERα but downregulated BRIP1 and PARP-1, similar to E_2_ (*p* < 0.05; Figure 5C–5I). Similarly, these positive effects of calycosin were efficiently suppressed by the additional administration of MPP or RP11-65M17.3 shRNA (*p* < 0.05 vs. 8 mg/kg calycosin alone), while E_2_-mediated proliferation was mainly blocked by MPP (*p* < 0.05 vs. 20 μg/kg E_2_ alone). Consistent with our *in vitro* studies, these results confirm the proposed role of the positive loop between ERα and RP11-65M17.3 in the calycosin-induced estrogenic effects on ECs.

### Comparison of calycosin and E_2_ treatment in tumor xenografts

**Figure 6:**
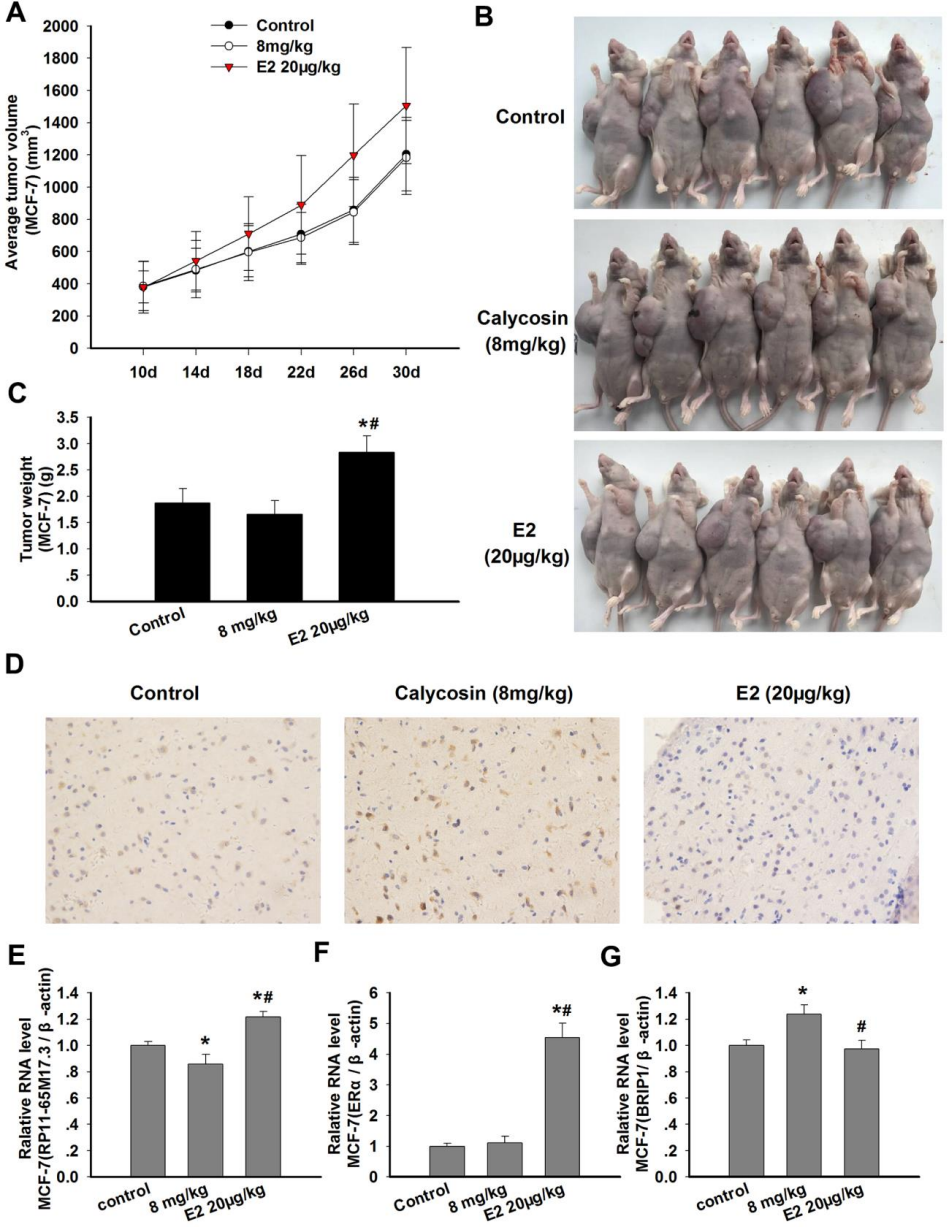
**Effects of calycosin on xenograft tumor growth and the expression levels of RP11-65M17.3, ERα and BRIP1 in tumor tissue.** (**A**–**C**) Nude mice bearing MCF-7-xenografted were treated for 20 days with calycosin (0 or 8 mg/kg) and 20 μg/kg E_2_. The tumor volume and tumor weight of the MCF-7 xenografts were measured (n = 6). (**D**) The tumor samples were subjected to IHC staining for BRIP1. (**E**–**G**) The mRNA expression levels of RP11-65M17.3, ERα and BRIP1 in the tumor tissues were determined using qRT-PCR, and β-actin served as the control. Representative data from three independent experiments are shown. *p < 0.05 vs. control; ^#^*p* < 0.05 vs. 8 mg/kg calycosin.

The ability of calycosin to stimulate the proliferation of BCCs *in vitro* prompted us to examine whether it would promote the growth of breast carcinoma *in vivo*. As shown in Figure 6A–6C, the greatest increase in tumor volume and weight was observed after treatment with E_2_, and this trend might be associated with the significant upregulation of RP11-65M17.3 and ERα expression in the xenografts (*p* < 0.05). In contrast, no significant increase was observed in the tumor volume and weight in the calycosin-treated group compared with those in the control group. The tumor volume and weight in the calycosin-treated group were lower than those in the control group, although there was no significant difference between these values. This observation may show that calycosin downregulated the RP11-65M17.3 levels and upregulated the BRIP1 levels *in vivo* (*p* < 0.05; Figure 6D–6G).

## DISCUSSION

In addition to its barrier function, the endothelium is involved in a variety of physiological and pathological processes. Impaired endothelial function is often considered a critical early event in the development of postmenopausal cardiovascular diseases [19]. By binding to ERs on vascular ECs, estrogen facilitates cell survival and maintains endothelial integrity [20]. However, the long-term clinical use of estrogen can increase the risk of breast cancer by promoting cell proliferation through ER-mediated effects [21]. Phytoestrogens, due to their structural similarity to mammalian estrogens, are suggested to exhibit biological activities similar to those of endogenous estrogens by binding to ERα or ERβ [22]. Moreover, it is well recognized that phytoestrogens are associated with a relatively low risk of cancer development compared with synthetic estrogen [23]. Therefore, many researchers have investigated the use of phytoestrogens as alternatives to HRT. Herein, we investigated the estrogenic effects of the calycosin phytoestrogen using an *in vitro* model of ERα-positive EC proliferation and *in vivo* models of OVX rats and xenograft mice, together with studies focusing on the mechanism of these effects via the ERα and lncRNA pathways.

In the present study, low concentrations of calycosin promoted cell proliferation in endothelial cell lines (HUVECs and HMEC-1 cells) and in BCCs expressing ERα (MCF-7 and T47D cells). One possible reason for this finding is that calycosin has estrogenic or antiestrogenic effects depending on the presence of estrogen. Herein, all the cell lines were exposed to a low-estrogen environment, and calycosin may act as an estrogen agonist. Calycosin also provides an appropriate *in vitro* model to mimic the postmenopausal hormonal status in women. Interestingly, further increasing the calycosin concentration to 20 μM still promoted the growth of HUVECs and HMEC-1 cells but exerted inhibitory effects on the two breast cancer lines. In contrast, E_2_-induced proliferative effects were observed in all four cell lines, and were much stronger in the MCF-7 and T47D cells. These results may further confirm that in the treatment of postmenopausal syndrome, the phytoestrogen calycosin is associated with a lower risk of postmenopausal breast cancer than E_2_. Consistent with these results, both calycosin and E_2_ caused an increase in the uterine weight and aortic EC proliferation in OVX rats, but only E_2_ stimulated MCF-7 xenograft tumor growth *in vivo*.

LncRNA screening identified the lncRNAs upregulated by calycosin (20 μM) in HUVECs. Among these lncRNAs, RP11-65M17.3 had the highest fold-change value and was selected to verify its role in the regulation of cell growth. We found that overexpressing RP11-65M17.3 increased proliferation of both ECs and BCCs, whereas silencing RP11-65M17.3 led to decreased cell survival, indicating that RP11-65M17.3 may function as a positive growth regulator in either normal or tumor cells. Our results further confirmed that the expression of BRIP1 was negatively regulated by RP11-65M17.3 in the four cell lines. We also found that overexpressing RP11-65M17.3 upregulated ERα, while inhibiting of ERα downregulated RP11-65M17.3. Therefore, we provide evidence that ERα-positive BCCs, HUVECs and HMEC-1 cells contain a positive feedback loop between RP11-65M17.3 and ERα, which result in the regulation of BRIP1 expression. This is also the first report of the involvement of the feedback loop in the proliferation of normal ECs or cancer cells.

Next, we found that calycosin-induced proliferation, RP11-65M17.3 and ERα upregulation, and BRIP1 downregulation were blocked by the inhibition of RP11-65M17.3 or ERα in HUVECs and HMEC-1 cells. These results suggested that the calycosin-induced proliferation of ECs is mediated by the activation of the RP11-65M17.3-ERα loop and by the negative regulation of BRIP1 expression. Unlike in ECs, in BCCs, calycosin increased ERα expression but reduced RP11-65M17.3 expression, followed by the upregulation of BRIP1 expression. Moreover, the inhibition of ERα significantly enhanced the antiproliferative effects of calycosin, whereas the silencing of RP11-65M17.3 expression reduced these effects. We speculate that, unlike that of ECs, the calycosin-induced growth of BCCs may be controlled, at least in part, by two independent pathways, namely, the activation of ERα signaling and inactivation of RP11-65M17.3 signaling, and the former is stronger than the latter. The fact that calycosin selectively stimulates the ERα-RP11-65M17.3 feedback loop in HUVECs and HMEC-1 cells may explain why the isoflavone stimulated proliferation in ECs more than that in BCCs. Regarding E_2_-induced proliferation in all the cell lines, the proliferative activity was attenuated after pretreatment with MPP, but not after pretreatment with RP11-65M17.3 shRNA, and an increase in ERα expression was observed. It was suggested that upregulated ERα, but not RP11-65M17.3, contributed to the growth of both normal and cancer cells induced by E_2_. Interestingly, an increase in RP11-65M17.3 expression and a decrease in BRIP1 expression were also observed in E_2_-treated cells. Considering the report that ER could regulate the expression of lncRNAs, we speculated that it upregulated ERα and promoted RP11-65M17.3 expression. However, the levels of RP11-65M17.3 were insufficient to account for cell proliferation.

The phosphatidylinositol-3-kinase (PI3K)/Akt and Raf/MEK/ERK signaling pathways play critical roles in cell proliferation [24–26]. Recent studies have suggested a positive correlation between ERα expression and ERK and Akt phosphorylation levels [27]. Additionally, BRIP1 was found to promote cell growth by negatively regulating pathways such as the Akt and ERK1/2 signaling pathways [28]. As expected, following the induction of ERα and inhibition of BRIP1, we found that calycosin increased the Akt and ERK1/2 phosphorylation levels in HUVECs and HMEC-1 cells. Conversely, in two calycosin-treated breast cancer cell lines, an increase in ERα expression was offset by a decrease in BRIP1 expression, leading to inactivation of Akt and ERK1/2. Pretreatment with MPP or RP11-65M17.3 shRNA attenuated these effects in both the endothelial cell lines. However, pretreatment with MPP reduced the effects of calycosin on the phosphorylation of Akt and ERK1/2 in BCCs, whereas RP11-65M17.3 shRNA enhanced the levels of p-Akt and p-ERK1/2. This finding again confirmed the involvement of the ERα- and RP11-65M17.3-mediated pathways in the calycosin-induced proliferation of BCCs. In the four E_2_-treated cell lines, with upregulated ERα expression and downregulated BRIP1 expression, the phosphorylation of Akt and ERK1/2 was increased, which could only be blocked by treatment with MPP.

Furthermore, in our previous study, poly(ADP-ribose) polymerase 1 (PARP-1), a chromatin-bound enzyme activated by DNA strand breaks, was proven to function as a downstream target of the PI3K/Akt and MAPK signaling pathways, and there was a negative correlation between these factors [29]. PARP-1 could be cleaved into 89-kD and 24-kD specific fragments, thus attenuating DNA repair and contributing to cell death [30]. We further showed that calycosin finally inhibited the cleavage of PARP-1 in normal ECs but promoted the cleavage of PARP-1 in BCCs. Similarly, increased cleavage of PARP-1 was observed in the four E_2_-treated cell lines. Additionally, these changes in PARP-1 were blocked by pretreatment with MPP or RP11-65M17.3 shRNA. These differential effects were also observed *in vivo*. Both calycosin and E_2_ caused an increase in the uterine weight and endometrial thickness in OVX rats, and these treatments increased the expression of ERα and RP11-65M17.3 but decreased the expression of BRIP1 and PARP-1. These effects were attenuated after pretreatment with MPP or RP11-65M17.3 shRNA. The *in vitro* and *in vivo* data suggest that the calycosin-induced proliferation of ECs involves the feedback loop between ERα and RP11-65M17.3. On the other hand, in the MCF-7 xenograft models, accompanied by increased tumor volume and weight, E_2_ significantly upregulated ERα and RP11-65M17.3 expression but exerted no effect on BRIP1 expression. However, calycosin downregulated RP11-65M17.3 expression and upregulated BRIP1 expression, in agreement with the *in vitro* data.

**Figure 7:**
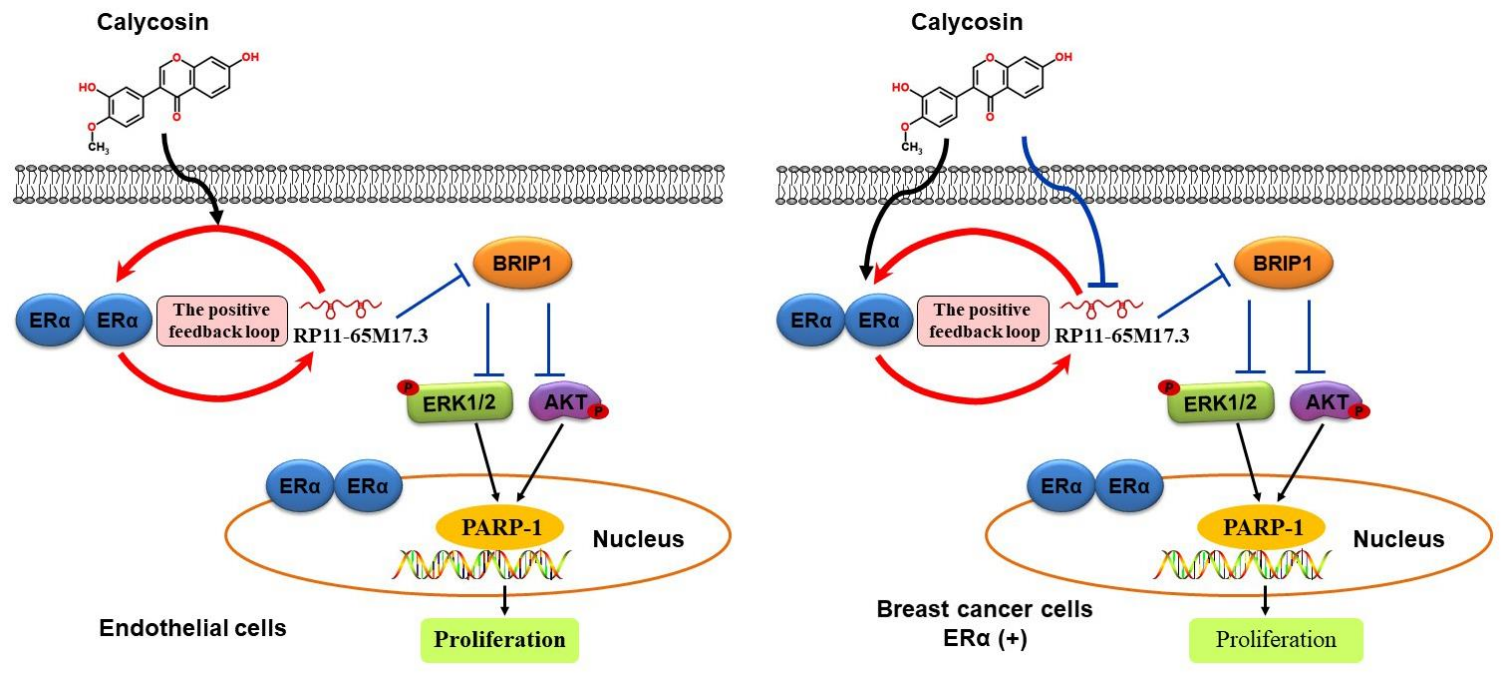
**Schematic illustration of the proposed mechanism by which calycosin promotes the proliferation of ECs and inhibits the growth of BCCs.** This selective activity of calycosin in ECs and BCCs depends on a feedback loop involving RP11-65M17.3 and ERα.

In summary, the present study provides *in vitro* and *in vivo* evidence that the phytoestrogen calycosin promotes the proliferation of ECs at low concentrations. Such action likely involves a positive feedback loop between RP11-65M17.3 and ERα, which then reduces BRIP1, activates the PI3K/Akt and ERK pathways, and finally inhibits PARP-1 cleavage (Figure 7). The fact that a low concentration of calycosin exerted inhibitory effects on the growth of MCF-7 or T47D BCCs may be mainly due to the inactivation of the RP11-65M17.3 pathway, suggesting that the long-term use of calycosin in treating postmenopausal syndrome could be associated with a lower risk of breast cancer than the long-term use of estrogen.

## MATERIALS AND METHODS

### Experimental design

This study comprised two parts: *in vitro* experiments in HUVECs and HMEC-1, MCF-7 and T47D cells, and *in vivo* experiments in OVX Wistar rats and nude mice bearing MCF-7-xenografts.

### Cell culture

HUVECs and HMEC-1 cells were cultured in F-12K medium and DMEM (Gibco, USA), respectively, containing the appropriate supplements and 10% fetal bovine serum (FBS; HyClone, USA) as previously described. The human breast cancer cell lines MCF-7 and T47D were grown in RPMI-1640 supplemented with 10% FBS and 1% penicillin/streptomycin (Invitrogen-Life Technologies, USA). All the cell lines were maintained at 37° C in a 5% CO_2_ humidified atmosphere.

Calycosin was purchased from Phytomarker (Tianjin, China), dissolved in dimethyl sulfoxide (DMSO), and then stored as a 200-mM stock solution at 4° C for further use. The cells were pretreated with phenol red-free MEM supplemented with 10% charcoal-dextran FBS (CDS; Invitrogen) for at least 4 days, followed by the addition of calycosin at different concentrations. 17β-Estradiol (E_2_; Sigma-Aldrich, USA) at a dose of 10 nM served as a positive control. For the inhibition assays, cells were pretreated with the ERα antagonist MPP and the plasmid constructs pCDNA3.1-RP11-65M17.3, pCDNA3.1(+) vector or RP11-65M17.3 shRNA (XuanC Bio, Guangdong, China) prior to incubation with E_2_ or calycosin (20 μM).

### Cell growth assay

HUVECs and HMEC-1, MCF-7 and T47D cells were treated with the indicated concentrations of each drug or vehicle for 12, 24 or 48 h. Next, cell survival was measured by the CCK-8 assay (Sigma, USA) and BrdU assay (Roche Applied Science, USA) according to the manufacturer’s instructions. For the colony formation assays, the cells were seeded in six-well plates. After 7, 14, and 21 days, the cell colonies were stained with 0.2% (w/v) crystal violet and then counted. The colony-forming rate was calculated by dividing the number of colonies per dish by the total number of cells plated per dish.

### Microarray of lncRNAs

Total RNA was extracted from HUVECs using the miRNeasy mini kit (Qiagen, USA) according to the manufacturer's protocol. RNA amplification and labeling were performed using the Amino Allyl MessageAmp II aRNA Amplification kit (Ambion, USA). Labeled cDNA was subjected to hybridization using the Human lncRNA OneArray Plus microarray (Phalanx Biotech Group, Taiwan), followed by scanning using an Agilent scanner (Agilent Technologies, USA). Agilent Feature Extraction software (version 11.0.1.1) was used to grid and extract the data.

### Gene function analysis

The predicted target genes of the differentially expressed lncRNAs were input into the Database for Annotation, Visualization and Integrated Discovery, which utilizes Gene Ontology (GO) analysis to analyze the molecular functions represented in the lncRNA profile (XuanC Bio). Furthermore, the potential functions of these differentially expressed lncRNAs in the pathways were determined by the Kyoto Encyclopedia of Genes and Genomes database.

### Luciferase reporter assay

To determine whether BRIP1 was the direct target of RP11-65M17.3, Lipofectamine 2000 (Invitrogen) was used to cotransfect HUVECs and HMEC-1 cells with a luciferase reporter plasmid (Promega) containing wild-type or mutated 3’-UTRs of BRIP1 with a plasmid encoding hsa-RP11-65M17.3 (XuanC Bio). Following transfection for 48 hours, the luciferase activity in each group was measured using a dual-luciferase assay system (Promega) and was normalized to the activity of Renilla luciferase.

### Real-time PCR assay

Total RNA was extracted from HUVECs and HMEC-1, MCF-7 and T47D cells using TRIzol reagent (Invitrogen) and then reverse transcribed into cDNA using the Revert Aid™ First Strand cDNA Synthesis Kit (Fermentas, USA). Real-time PCR was carried out using primers specific for ERα, RP11-65M17.3, BRIP1 and poly (ADP-ribose) polymerase 1 (PARP-1) and the BioRad iQ5 real-time PCR system. β-Actin and U6 snRNA served as the internal controls for normalization.

### Western blot assay

Proteins were extracted from HUVECs and HMEC-1, MCF-7 and T47D cells, separated by SDS-polyacrylamide gel electrophoresis, and transferred to polyvinylidene difluoride (PVDF) membranes. After blocking with 5% nonfat milk, the membranes were probed with primary antibodies against ERα, BRIP1, ERK1/2, Akt, p-ERK1/2, p-Akt, PARP-1 and β-actin. All the antibodies were purchased from Santa Cruz Biotechnology (Santa Cruz, USA). For quantification, the densities of the specific bands were measured and normalized to the loading control band.

### Ovariectomy

Adult female Wistar rats were housed in a temperature- and humidity-controlled room with a 12-h light/dark cycle. The rats were anesthetized with 3% sodium phenobarbital, and underwent ovariectomy surgery (OVX) or sham operation (sham). Five days later, the rats were randomly divided into seven groups (n=6 per group): sham controls, OVX rats, OVX rats receiving intraperitoneal (ip) injection of calycosin (8 mg/kg/day), OVX rats receiving injection of calycosin (8 mg/kg/day, ip) and MPP (5 mg/kg/day, ip), OVX rats receiving injection of calycosin (8 mg/kg/day, ip) and RP11-65M17.3 shRNA, OVX rats receiving injection of E_2_ (20 μg/kg/day, ip), OVX rats receiving injection of E_2_ (20 μg/kg/day, ip) and MPP (1 mg/kg/day, ip), and OVX rats receiving injection of E_2_ (20 μg/kg/day, ip) and RP11-65M17.3 shRNA. After 20 days of treatment, the rats were sacrificed to measure the uterine weight, and the chest was quickly opened. With its adherent connective tissue carefully separated and removed, the thoracic aorta was immersed in sterile PBS containing an RNase inhibitor. Aortic ECs were harvested by gentle scraping with a surgical blade and stored at -80° C. The uterine tissues were stained with hematoxylin and eosin (HE) staining as previously described, and examined by light microscopy. The expression levels of ERα, RP11-65M17.3, BRIP1, and PARP-1 in the aortic ECs were determined using qRT-PCR and Western blotting.

### Tumor xenografts

The nude mice were subcutaneously injected with 1×10^7^ MCF-7 cells. When the tumor approached 2 cm in diameter, it was cut into pieces of approximately 1 mm × 1 mm × 1 mm. These pieces were subcutaneously implanted into the right armpit of recipient nude mice. When the tumors were established and had reached a size of 0.2 cm^3^, the nude mice were treated with calycosin (8 mg/kg/day, ip) or E_2_ (20 μg/kg/day, ip) for 20 days. The nude mice were examined every 4 days, and tumor growth was recorded. After 30 days of treatment, the tumors were harvested, and the expression levels of ERα, RP11-65M17.3 and BRIP1 were determined using qRT-PCR. All the animal experiments were approved by the Institutional Animal Research Ethics Committee of Guilin Medical University.

### Tumor samples and histological examination

Sections of paraffin-embedded tumor samples were subjected to IHC staining. For IHC, the sections were deparaffinized, hydrated, and immersed in 1% hydrogen peroxide in methanol for 30 minutes to block the endogenous peroxidase activity. The sections were incubated with a rabbit anti-BRIP1 polyclonal antibody (Abcam, Cambridge, Cambridgeshire, UK; diluted 1:250) overnight at 4° C. After washing with PBS, the sections were incubated with a biotinylated secondary antibody (diluted 1:100) for 30 minutes at 37° C, followed by exposure to horseradish peroxidase-conjugated goat anti-rabbit IgG for 20 minutes at 37° C. The immunoreactive signal was visualized by the DAB detection system.

### Statistical analysis

The data are presented as the mean ± standard deviation (SD). Comparisons between groups were performed by one-way ANOVA using Statistical Package for Social Sciences (SPSS) 13.0 software (SPSS, USA). A value of *p* < 0.05 was considered to be statistically significant.

## References

[1] Wong KL, Lai YM, Li KW, Lee KF, Ng TB, Cheung HP, Zhang YB, Lao L, Wong RN, Shaw PC, Wong JH, Zhang ZJ, Lam JK, et al. A Novel, Stable, Estradiol-Stimulating, Osteogenic Yam Protein with Potential for the Treatment of Menopausal Syndrome. Sci Rep. 2015; 5:10179. 10.1038/srep10179 26160710 PMC5155516

[2] Diczfalusy E. The third age, the third world and the third millennium. Contraception. 1996; 53:1–7. 10.1016/0010-7824(95)00258-8 8631183

[3] Rosano GM, Spoletini I, Vitale C. Cardiovascular disease in women, is it different to men? the role of sex hormones. Climacteric. 2017; 20:125–28. 10.1080/13697137.2017.1291780 28286991

[4] Filipe C, Lam Shang Leen L, Brouchet L, Billon A, Benouaich V, Fontaine V, Gourdy P, Lenfant F, Arnal JF, Gadeau AP, Laurell H. Estradiol accelerates endothelial healing through the retrograde commitment of uninjured endothelium. Am J Physiol Heart Circ Physiol. 2008; 294:H2822–30. 10.1152/ajpheart.00129.2008 18441207

[5] Lan XF, Zhang XJ, Lin YN, Wang Q, Xu HJ, Zhou LN, Chen PL, Li QY. Estradiol regulates txnip and prevents intermittent hypoxia-induced vascular injury. Sci Rep. 2017; 7:10318. 10.1038/s41598-017-10442-7 28871193 PMC5583380

[6] Tormey SM, Malone CM, McDermott EW, O’Higgins NJ, Hill AD. Current status of combined hormone replacement therapy in clinical practice. Clin Breast Cancer. 2006 (Suppl 2); 6:S51–57. 10.3816/cbc.2006.s.004 16595027

[7] Li SH, Liu XX, Bai YY, Wang XJ, Sun K, Chen JZ, Hui RT. Effect of oral isoflavone supplementation on vascular endothelial function in postmenopausal women: a meta-analysis of randomized placebo-controlled trials. Am J Clin Nutr. 2010; 91:480–86. 10.3945/ajcn.2009.28203 19923372

[8] Basu P, Maier C. Phytoestrogens and breast cancer: *in vitro* anticancer activities of isoflavones, lignans, coumestans, stilbenes and their analogs and derivatives. Biomed Pharmacother. 2018; 107:1648–66. 10.1016/j.biopha.2018.08.100 30257383

[9] Wood CE, Register TC, Franke AA, Anthony MS, Cline JM. Dietary soy isoflavones inhibit estrogen effects in the postmenopausal breast. Cancer Res. 2006; 66:1241–49. 10.1158/0008-5472.CAN-05-2067 16424064

[10] Chen J, Zhang X, Wang Y, Ye Y, Huang Z. Differential ability of formononetin to stimulate proliferation of endothelial cells and breast cancer cells via a feedback loop involving MicroRNA-375, RASD1, and ERα. Mol Carcinog. 2018; 57:817–30. 10.1002/mc.22531 29722068

[11] Tang JY, Li S, Li ZH, Zhang ZJ, Hu G, Cheang LC, Alex D, Hoi MP, Kwan YW, Chan SW, Leung GP, Lee SM. Calycosin promotes angiogenesis involving estrogen receptor and mitogen-activated protein kinase (MAPK) signaling pathway in zebrafish and HUVEC. PLoS One. 2010; 5:e11822. 10.1371/journal.pone.0011822 20686605 PMC2912279

[12] Chen J, Liu L, Hou R, Shao Z, Wu Y, Chen X, Zhou L. Calycosin promotes proliferation of estrogen receptor-positive cells via estrogen receptors and ERK1/2 activation *in vitro* and *in vivo*. Cancer Lett. 2011; 308:144–51. 10.1016/j.canlet.2011.04.022 21612861

[13] Yoon JH, Abdelmohsen K, Gorospe M. Functional interactions among microRNAs and long noncoding RNAs. Semin Cell Dev Biol. 2014; 34:9–14. 10.1016/j.semcdb.2014.05.015 24965208 PMC4163095

[14] Peng J, Zhang L, Yuan C, Zhou L, Xu S, Lin Y, Zhang J, Yin W, Lu J. Expression profile analysis of long noncoding RNA in ER-positive subtype breast cancer using microarray technique and bioinformatics. Cancer Manag Res. 2017; 9:891–901. 10.2147/CMAR.S151120 29276409 PMC5733923

[15] Hah N, Kraus WL. Hormone-regulated transcriptomes: lessons learned from estrogen signaling pathways in breast cancer cells. Mol Cell Endocrinol. 2014; 382:652–64. 10.1016/j.mce.2013.06.021 23810978 PMC3844033

[16] Watt LF, Panicker N, Mannan A, Copeland B, Kahl RG, Dun MD, Young B, Roselli S, Verrills NM. Functional importance of PP2A regulatory subunit loss in breast cancer. Breast Cancer Res Treat. 2017; 166:117–31. 10.1007/s10549-017-4403-5 28744751

[17] Cantor SB, Guillemette S. Hereditary breast cancer and the BRCA1-associated FANCJ/BACH1/BRIP1. Future Oncol. 2011; 7:253–61. 10.2217/fon.10.191 21345144 PMC3109611

[18] Perrotti D, Neviani P. Protein phosphatase 2A: a target for anticancer therapy. Lancet Oncol. 2013; 14:e229–38. 10.1016/S1470-2045(12)70558-2 23639323 PMC3913484

[19] Rosano GM, Vitale C, Fini M. Hormone replacement therapy and cardioprotection: what is good and what is bad for the cardiovascular system? Ann N Y Acad Sci. 2006; 1092:341–48. 10.1196/annals.1365.031 17308159

[20] Khalil RA. Estrogen, vascular estrogen receptor and hormone therapy in postmenopausal vascular disease. Biochem Pharmacol. 2013; 86:1627–42. 10.1016/j.bcp.2013.09.024 24099797 PMC3840081

[21] Bernstein L. The risk of breast, endometrial and ovarian cancer in users of hormonal preparations. Basic Clin Pharmacol Toxicol. 2006; 98:288–96. 10.1111/j.1742-7843.2006.pto_277.x 16611204

[22] Liu HX, Wang Y, Lu Q, Yang MZ, Fan GW, Karas RH, Gao XM, Zhu Y. Bidirectional regulation of angiogenesis by phytoestrogens through estrogen receptor-mediated signaling networks. Chin J Nat Med. 2016; 14:241–54. 10.1016/S1875-5364(16)30024-3 27114311

[23] Leclercq G, Jacquot Y. Interactions of isoflavones and other plant derived estrogens with estrogen receptors for prevention and treatment of breast cancer-considerations concerning related efficacy and safety. J Steroid Biochem Mol Biol. 2014; 139:237–44. 10.1016/j.jsbmb.2012.12.010 23274118

[24] Li Q, Wang C, Wang Y, Sun L, Liu Z, Wang L, Song T, Yao Y, Liu Q, Tu K. HSCs-derived COMP drives hepatocellular carcinoma progression by activating MEK/ERK and PI3K/AKT signaling pathways. J Exp Clin Cancer Res. 2018; 37:231. 10.1186/s13046-018-0908-y 30231922 PMC6146743

[25] Lu H, Guo Y, Gupta G, Tian X. Mitogen-activated protein kinase (MAPK): new insights in breast cancer. J Environ Pathol Toxicol Oncol. 2019; 38:51–59. 10.1615/JEnvironPatholToxicolOncol.2018028386 30806290

[26] Dong Q, Yang B, Han JG, Zhang MM, Liu W, Zhang X, Yu HL, Liu ZG, Zhang SH, Li T, Wu DD, Ji XY, Duan SF. A novel hydrogen sulfide-releasing donor, HA-ADT, suppresses the growth of human breast cancer cells through inhibiting the PI3K/AKT/mTOR and Ras/Raf/MEK/ERK signaling pathways. Cancer Lett. 2019; 455:60–72. 10.1016/j.canlet.2019.04.031 31042588

[27] Wang C, Xu CX, Bu Y, Bottum KM, Tischkau SA. Beta-naphthoflavone (DB06732) mediates estrogen receptor-positive breast cancer cell cycle arrest through AhR-dependent regulation of PI3K/AKT and MAPK/ERK signaling. Carcinogenesis. 2014; 35:703–13. 10.1093/carcin/bgt356 24163404 PMC3941744

[28] Schulten HJ, Bangash M, Karim S, Dallol A, Hussein D, Merdad A, Al-Thoubaity FK, Al-Maghrabi J, Jamal A, Al-Ghamdi F, Choudhry H, Baeesa SS, Chaudhary AG, Al-Qahtani MH. Comprehensive molecular biomarker identification in breast cancer brain metastases. J Transl Med. 2017; 15:269. 10.1186/s12967-017-1370-x 29287594 PMC5747948

[29] Chen J, Hou R, Zhang X, Ye Y, Wang Y, Tian J. Calycosin suppresses breast cancer cell growth via ERβ-dependent regulation of IGF-1R, p38 MAPK and PI3K/Akt pathways. PLoS One. 2014; 9:e91245. 10.1371/journal.pone.0091245 24618835 PMC3949755

[30] Rajawat J, Shukla N, Mishra DP. Therapeutic Targeting of Poly(ADP-Ribose) Polymerase-1 (PARP1) in Cancer: Current Developments, Therapeutic Strategies, and Future Opportunities. Med Res Rev. 2017; 37:1461–91. 10.1002/med.21442 28510338

