# Correction for: Calycosin stimulates the proliferation of endothelial cells, but not breast cancer cells, via a feedback loop involving RP11-65M17.3, BRIP1 and ERα

**DOI:** 10.18632/aging.203953

**Published:** 2022-03-14

**Authors:** Yong Wang, Wei Xie, Mengyue Hou, Jing Tian, Xing Zhang, Qianyao Ren, Yue Huang, Jian Chen

**Affiliations:** 1 Key Laboratory of Tumor Immunology and Microenvironmental Regulation of Guangxi, Guilin Medical University, Guilin 541004, Guangxi, China; 2 Department of Physiology, Guilin Medical University, Guilin 541004, Guangxi, China; 3 Department of Breast and Thyroid Surgery, First Affiliated Hospital of Guilin Medical University, Guilin 541001, Guangxi, China

**Keywords:** calycosin, postmenopausal, endothelial cells, breast cancer, RP11-65M17.3

Original article: Aging. 2021; 13:11026–11042.  . https://doi.org/10.18632/aging.202641

**This article has been corrected:** In the new **Figure 5**, the authors replaced **Figure 5B**, where they accidently mislabeled the “E2” and “E2+inhibitor” groups and mistakenly used partially duplicated pictures. The new **Figure 5B** contains a new image for the “E2+inhibitor” group from the original set of experiments. The authors also provided all the original HE staining pictures for this manuscript. This correction does not change the content of the publication and does not affect the conclusion of this research.

New **Figure 5** is presented below.

**Figure 5:**
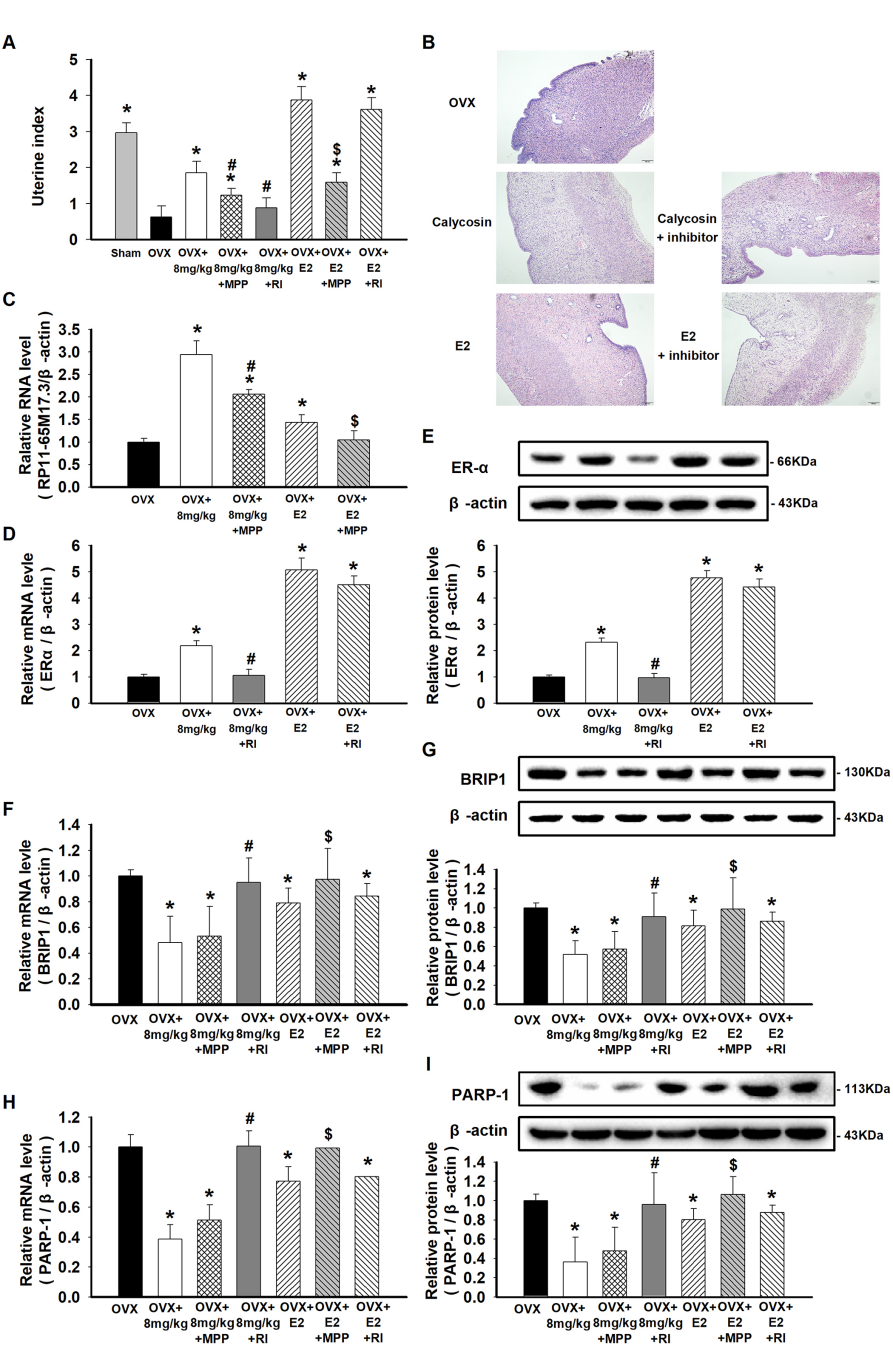
**Effects of calycosin on OVX rats and the activation of the RP11-65M17.3-ERα loop in aortic ECs**. (**A**) OVX rats were treated for 20 days with calycosin (0 or 8 mg/kg), 8 mg/kg calycosin and 5 mg/kg MPP, 8 mg/kg calycosin and RP11-65M17.3 shRNA, 20 μg/kg E_2_, 20 μg/kg E_2_ and 5 mg/kg MPP, 20 μg/kg E_2_ and RP11-65M17.3 shRNA. The uterine index was calculated by the percentage of the uterus weight relative to the body weight. (**B**) The uterine tissues were stained with HE. (**C**–**I**) The levels of RP11-65M17.3, ERα, BRIP1 and PARP-1 in aortic ECs were determined using qRT-PCR or Western blotting. Representative data from three independent experiments are shown. *p < 0.05 vs. OVX; ^#^*p* < 0.05 vs. 8 mg/kg calycosin; ^$^*p* < 0.05 vs. 20 μg/kg E_2._

